# Swimming in a school shelters fish from turbulence

**DOI:** 10.1371/journal.pbio.3002677

**Published:** 2024-06-07

**Authors:** Tyson L. Hedrick

**Affiliations:** University of North Carolina at Chapel Hill, Department of Biology, Chapel Hill, North Carolina, United States of America

## Abstract

Much has been written about the energetic effects of animals moving in schools or flocks, but experimental results are few and often ambiguous. This Primer explores a new study in PLOS Biology which shows that schooling greatly reduces the cost of transport for fish in turbulent flow.

## Teaser

Much has been written about the energetic effects of animals moving in schools or flocks, but experimental results are few and often ambiguous. A new study in *PLOS Biology* shows that schooling greatly reduces the cost of transport for fish in turbulent flow.

Animals move together in groups for many reasons, from safety in numbers to pooling navigational knowledge [1,2]. However, for animals that swim or fly, moving together also has immediate fluid-dynamic consequences for the group members, potentially reducing their energetic cost of transport [3,4]. This attractive hypothesis has been the basis for many modeling and computational investigations, but the difficulty of making metabolic or biomechanical measurements from several animals moving together has made support from experimental data hard to come by. Furthermore, even when they exist, these data are often proxies of energetic expenditure such as heart rate [5], tailbeat frequency [6], or flapping phase [7] and cannot provide an unambiguous demonstration of energy savings.

**Fig 1:**
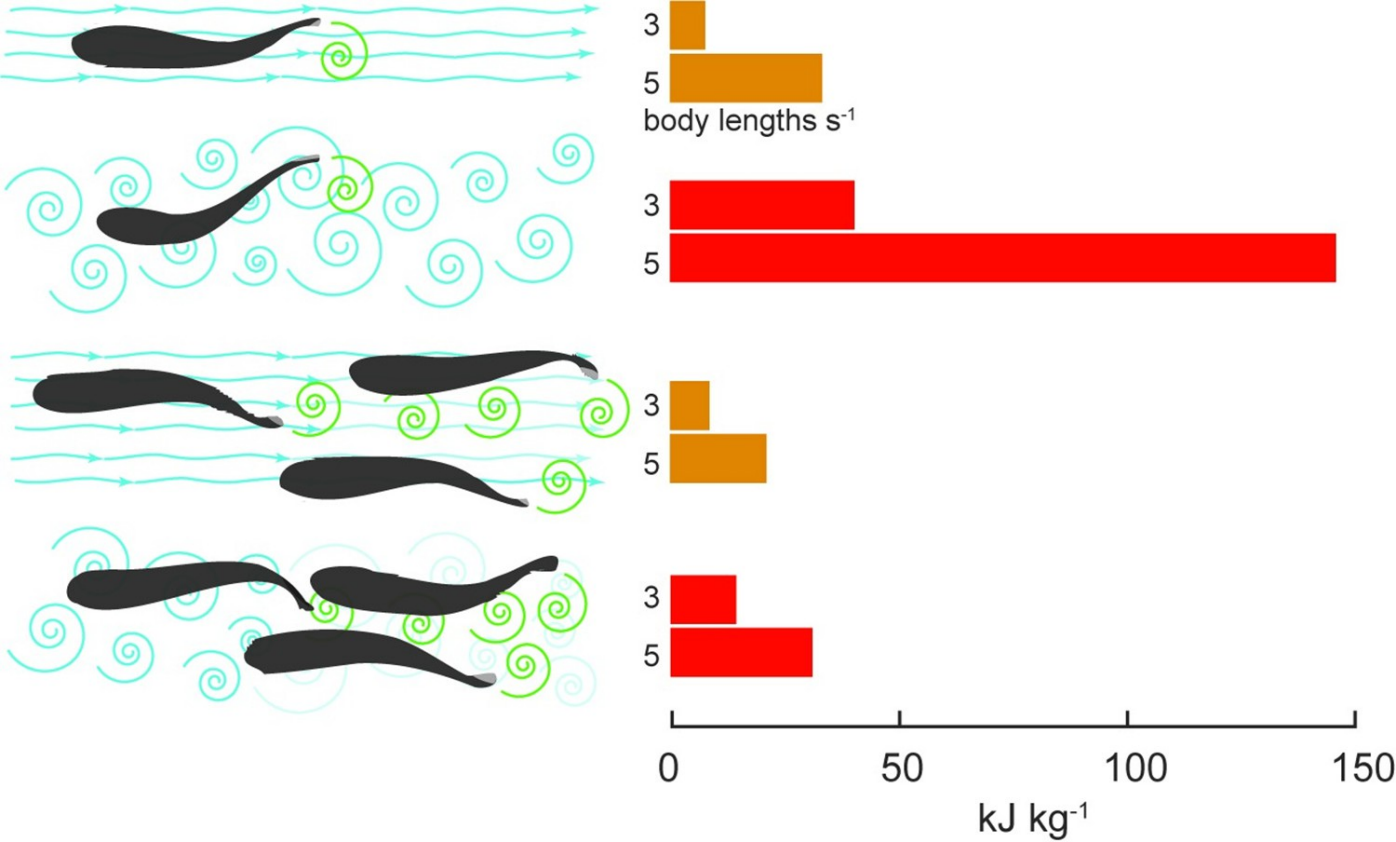
Cost of swimming at 2 speeds in 4 conditions. Zhang and colleagues [8] investigated how swimming in a school vs. solo affects cost of movement in turbulent and smooth flows. Schooling provided a mix of costs and benefits in smooth flows, but dramatically reduced costs in turbulent flows, suggesting that schooling provides a “turbulence shelter” where the close-packed schooling fish shield one another from external turbulence. This schematic figure shows the flow and grouping conditions on the left side including environmental flows (blue) and fish wakes (green), and the cost of movement on the right side. Data are from Zhang and colleagues [8], who provide results from a wider range of speeds and as cost of transport (kJ kg^−1^ km^−1^) in addition to total cost of swimming (kJ kg^−1^) as shown here. Fish were tested swimming solo and in groups of 8.

Zhang and colleagues’ new study in *PLOS Biology* [8] directly measured oxygen consumption and therefore energy expenditure from swimming fish, showing a noticeable energetic benefit to schooling over solitary swimming in smooth (i.e., laminar) flow conditions, especially at high speed. However, it also added a further twist by looking at how swimming in a school affects the cost of movement in turbulent flows. This revealed a much large beneficial effect for schooling under arguably more naturalistic conditions (Fig 1). In fact, in their turbulent flow experiment fish swimming as a group of 8 used only 21% as much energy compared to a solo fish swimming in the same flow conditions (3.7 versus 17.5 kJ km^−1^ kg^−1^) at 6 body lengths per second—an extraordinary improvement with widespread implications for understanding collective animal movement.

In Zhang and colleagues’ experiment Giant Danio, a small tropical fish found in shoals (i.e., schools) in nature were tested in 4 conditions in a laboratory swim tunnel (i.e., aquatic treadmill): swimming solo in laminar or turbulent flow, and swimming as a school of 8 in laminar or turbulent flow. The swim-tunnel apparatus measured oxygen consumption by the fish and used high-speed video to record the fish positions and kinematic features such as tailbeat frequency. As expected, in the solo swim tests turbulence substantially increased the cost of movement. For example, at 6 body lengths per second, cost of transport for an individual fish in turbulent flow was about 3-fold higher than in laminar flow (4.3 versus 17.5 kJ km^−1^ kg^−1^). This increase in swimming cost was coincident with an increase in tailbeat frequency and amplitude, biomechanical indicators of increased swimming effort. The effect of turbulence was also apparent in another way—when swimming in smooth flows, individual fish have a characteristic and consistent head oscillation amplitude and frequency due to head motion incorporated into the traveling body wave that produces fluid propulsion. When swimming in turbulent flow this consistent oscillation broke down as the fish were also pushed about by the environmental turbulence, increasing the range of oscillation frequencies and amplitudes.

Compared to fish swimming solo, fish swimming as a school at moderate to high speeds in a smooth flow had somewhat lower costs, e.g., 1.7 versus 2.4 kJ km^−1^ kg^−1^ at 4 body lengths per second, with the advantage of schooling increasing further at higher speeds. Swimming as a school also produced a different signature in the head oscillation amplitude and frequency from solo swimming, indicating that—as expected—schooling produces some internal vortices as the wakes of upstream fish interact with their downstream neighbors, but apparently to the net benefit of the schooling fish as evidenced by their lower cost of movement at faster swimming speeds (Fig 1).

The biggest surprise in the results was found when the fish were tested as a school in turbulent flow. The cost of movement of the schooling fish in turbulence did increase slightly, e.g., from 1.7 to 2.2 kJ km^−1^ kg^−1^ at 4 body lengths per second but remained well below that of a solo fish in turbulence at the same speed (9.4 kJ km^−1^ kg^−1^), demonstrating a more than 4-fold cost of transport savings for schooling versus solo swimming under these conditions. Head oscillation amplitude and frequency was again altered, with a wider range of frequencies appearing but the total oscillation amplitude remaining consistent among schooling results from smooth and turbulent flow conditions. Interestingly, the overall school shape also changed in turbulent compared to smooth flow, with the fish packing closer together and adopting a more elongate school shape.

The changes to head oscillation characteristics and school shape in turbulent flow help support Zhang and colleagues’ overall contention—that a school provides a shelter from environmental turbulence. The evidence available thus far consistent with that hypothesis, but like any interesting research result, the current study raises many new questions. For example, Zhang and colleagues used a fixed-size, passive grid inserted into the flow to generate turbulence with a characteristic size similar to fish body dimensions, and it will be interesting see over what turbulence size scales and magnitudes the schooling “turbulence shelter” is most effective, along with better measurement of the turbulence experienced by fish in different natural circumstances. Furthermore, the exact mechanism that produces the turbulence shelter is uncertain as are the details of the flow environment inside the school, and what (if any) positioning relationships are required among the fish in the school to take advantage of the shelter. The school increases in density and changes in shape in turbulence, but whether these are necessary components to the effect or merely enhancements of it remains to be determined. It is also unknown whether similar effects might exist in bird flocks, where some evidence points toward cluster flocks increasing flight costs [9], but also having potentially beneficial internal structure [10]. Lastly, the implications of Zhang and colleagues’ results for natural movement patterns require further investigation. Although schooling provided highly effective protection from the effect of turbulence at higher speeds, the very lowest cost of transport in both turbulent and smooth flow was found for solo fish swimming slowly, at about 2 body lengths per second. Thus, schooling was not universally advantageous, even in the turbulent flows examined here.

Despite these many follow-up questions, Zhang and colleagues provide an exceptionally clear demonstration of how collective locomotion shapes the costs of movement. Here, the effects are substantial enough that they may be fundamental to the capability of different organisms to effectively forage, migrate, escape predation, and accomplish other movement-related tasks.
